# The Effects of Cognitive Ability, Mental Health, and Self-Quarantining on Functional Ability of Older Adults During the COVID-19 Pandemic: Results From the Canadian Longitudinal Study on Aging

**DOI:** 10.1177/08919887231218755

**Published:** 2023-12-20

**Authors:** Madeline A. Gregory, Morgan J. Schaeffer, Jennifer T. H. Reeves, Lauren E. Griffith, Christina Wolfson, Nicole E. Basta, Jacqueline M. McMillan, Susan Kirkland, Parminder Raina, Theone S. E. Paterson, on behalf of the Canadian Longitudinal Study on Aging (CLSA) Team

**Affiliations:** 1 Department of Psychology, 8205 University of Victoria, Victoria, BC, Canada; 2 Department of Health Research Methods, Evidence, and Impact, Faculty of Health Sciences, 3710 McMaster University, Hamilton, ON, Canada; 3 McMaster Institute for Research on Aging, 3710 McMaster University, Hamilton, ON, Canada; 4 Department of Epidemiology, Biostatistics and Occupational Health, School of Population and Global Health, 5620 McGill University, Montreal, QC, Canada; 5 Department of Medicine, 70401 University of Calgary Cumming School of Medicine, Calgary, AB, Canada; 6 Department of Community Health & Epidemiology and Division of Geriatric Medicine, 3688 Dalhousie University, Halifax, NS, Canada; 7 Neuropsychology and Cognitive Health, Baycrest Health Sciences Centre, Toronto, ON, Canada

**Keywords:** Canadian longitudinal study on aging, anxiety, depression, COVID-19, functional ability, mental health, cognition

## Abstract

**Objectives:**

Determine whether levels of anxiety and depression, cognitive ability, and self-quarantining during and prior to the pandemic predict decreases in perceived functional ability.

**Design and Setting:**

Longitudinal data collected from the Canadian Longitudinal Study on Aging (CLSA) COVID-19 Questionnaire Study (2020) and core CLSA study (Follow-Up 1; 2014-2018).

**Participants:**

17 541 CLSA participants.

**Measurements:**

Self-quarantining behaviours from questionnaires administered at Baseline (April 2020), Monthly, and Exit (December 2020) time points of the CLSA COVID-19 Questionnaire Study, levels of anxiety and depression at Baseline, perceived change in functional ability at Exit, and performance on neuropsychological tests (Rey Auditory Verbal Learning Task, Mental Alternation Task, Animal Fluency Test) and functional ability (Older Americans Resources and Services [OARS] Multidimensional Assessment Questionnaire) from the core CLSA study.

**Results:**

Greater cognitive ability pre-pandemic (*B* = −.003, *P* < .01), higher levels of anxiety (*B* = −.024, *P* < .01) and depressive symptoms (*B* = −.110, *P* < .01) at Baseline, and higher frequency of engaging in self-quarantining throughout the COVID-19 survey period (*B* = −.098, *P* < .01) were associated with perceived loss in functional ability at Exit. Self-quarantining behaviour was associated with perceived loss in functional ability only at average and high levels of depressive symptoms (*B* = −.013, *P* < .01).

**Conclusions:**

Older adults with higher cognitive and lower functional ability prior to the pandemic were at greater risk of decreased perceived functional ability during the first year of the pandemic, as were those who experienced greater levels of anxiety and depressive symptoms during the pandemic. Strategies/interventions to preserve functional ability in older adults with cognitive independence prior to future pandemics are warranted.

## Introduction

The Coronavirus Disease 2019 (COVID-19) pandemic has had widespread effects on older individuals’ daily lives. To reduce the spread of the virus, governments have implemented public health measures involving policies such as physical distancing, mask wearing, and quarantining when necessary.^1^ To align with these policies, the behaviours of Canadians have changed. For example, many individuals have limited their in-person interactions with their family and friends to abide by physical distancing recommendations or requirements and have decreased how often they leave the house for non-essential errands. Moreover, it is likely that the COVID-19 pandemic has affected the perceived functional ability of older adults.

Functional ability, which is an individual’s capacity to engage in their typical activities of daily living, often decreases with age,^2^ and can be a concern among older adults. Functional ability can be measured using objective performance-based tasks (e.g., range of motion, balance, efficacy at performing an activity) or subjective report measures (e.g., rating scales, interviews). A limitation of subjective tests is that older adults may not have accurate perception of their own capabilities (particularly if they experience fluctuating levels of physical symptoms such as pain).^3^ However, older adults’ perceptions of their own functional ability have been found to be more accurate than that of family members and physicians, making self-perception of ability a valuable indicator of functional ability where performance-based measures are unavailable.^4^ Predictors of functional dependence in community-dwelling older adults include poor physical health such as frailty, presence of chronic conditions, lack of exercise, and poor diet, declining cognitive status, and mental illness.^5,6^ In assisted-living settings, global cognition and psychiatric symptoms were found to explain 25% of the variance in functional dependence for individuals without dementia while chronic conditions and diabetes played a larger role in predicting level of functional dependence in individuals with dementia.^7^ As such, cognitive and psychological/emotional factors may play a more important role than physical functioning in functional dependence of older adults without cognitive syndromes.

Several studies have reported lower levels of physical activity among older adults during the COVID-19 pandemic, including a recent study which reported that Canadian older adults with a confirmed, probable, or suspected diagnosis of COVID-19 had nearly 2-fold higher odds of worsening mobility and functional ability compared to their peers who did not contract the virus.^8^ Even among those who have not been infected with the virus, changes in behaviour during the COVID-19 pandemic may also have implications for functional ability. Likely due to a desire to reduce their daily activities outside of their household (e.g., decreased shopping as family members take over responsibilities), older adults have reported declines in their engagement in physical activity, with individuals aged 55 to 64 reporting engaging in on average 47 minutes less moderate intensity physical activity than before the pandemic.^9^ The COVID-19 pandemic may have promoted a negative cycle of declining functional ability, with decreased activity and increased sedentary behaviours due to the pandemic leading to decreased muscle power and function, ultimately reducing functional ability.^10^ In addition to objective declines in physical ability, the pandemic may also influence perceived functional decline, or an individual’s perception of their own day-to-day functional abilities. As functional ability is often self-reported, evidence suggests that it may be affected by many other factors associated with the COVID-19 pandemic and quarantine, including depression.^11^

The COVID-19 pandemic has also had substantial impacts on mental health.^12^ Mental health has implications for the functional ability of older adults, as individuals who report worse mental health (e.g., more anxiety and depressive symptoms) typically experience a greater decline in functional ability over time.^13^ It is unknown how the declines in mental health during the pandemic have affected the functional ability of older individuals. However, as the proportion of Canadians who report their level of anxiety symptoms to be either high or extremely high has quadrupled compared to pre-pandemic times, while reports of high levels of depressive symptoms have doubled,^14^ it is likely that more older adults will begin to perceive their functional ability as declining as the pandemic progresses. Additionally, adhering to social distancing policies has been associated with reported decreases in psychological wellbeing,^15^ indicating possible relations between mental health and self-quarantining behaviour and their combined impacts on the overall functioning of older adults.

When considering mental health and functional ability in older adults, it is also important to account for the role of cognitive decline. Previous work has found associations between cognitive decline and both depression^16^ and anxiety.^17^ Moreover, cognitive ability may have particularly salient interactions with mental health in the context of the COVID-19 pandemic. For example, older adults who are experiencing cognitive decline may become more anxious, angry, stressed, or agitated during periods of social isolation.^18^

There is also an established independent relationship between functional and cognitive decline; cognitive and functional decline both influence the development of one another, with greater cognitive decline associated with greater functional decline and vice versa.^19^ Therefore, cognitive ability prior to the onset of the pandemic may have an important influence on changes to perceived functional ability during the pandemic. To better support older adults during this and future pandemics, it is important to examine the implications of mood, cognition, and behaviour related to the COVID-19 pandemic.

### Aims of the Present Study

This study aimed to identify whether and how the onset of the COVID-19 pandemic affected older adults’ perceptions of their change in functional ability, and the influence of cognitive ability, mental health, and pandemic-related behaviours on these perceptions. Based on the previous findings highlighted above regarding relationships among functional ability, physical health, cognition, and mental health, we hypothesized that lower pre-pandemic cognitive ability and higher functional ability prior to the pandemic, as well as greater levels of anxiety and depressive symptoms at the start of the pandemic, would be associated with perceived decreases in functional ability since the beginning of the pandemic. We also hypothesized that greater frequency of self-quarantining would be associated with perceived decreases in functional ability. Lastly, we hypothesized that there would be interactions between self-quarantining and anxiety and depression such that the relationship between frequency of engaging in self-quarantining behaviour and perceived change in functional ability would be stronger for those with higher levels of depression and anxiety at the start of the pandemic.

#### Methods

##### Participants

The data for this study were from the Canadian Longitudinal Study on Aging (CLSA).^20^ The CLSA is a large national study that included 51 338 individuals between the ages of 45 and 85 at recruitment. CLSA baseline demographic, clinical, and cognitive information were collected between 2011 and 2015 with the first follow-up assessment occurring approximately three years after baseline (FUP1, 2014-2018). The core CLSA study consists of two cohorts: a Tracking cohort and a Comprehensive cohort. The Tracking cohort consists of 21 241 participants randomly selected from across Canada who provide information through telephone interviews. The Comprehensive cohort consists of 30 097 participants who lived within a 25-km radius of 1 of the 11 CLSA data collection sites across Canada at baseline. Participants in this cohort undergo an in-person, in-home interview, extensive physical assessments, and provide biological samples at each data collection site visit.

In response to the COVID-19 pandemic, the CLSA launched the COVID-19 Questionnaire Study which aimed to help understand the impacts of the COVID-19 pandemic on older adults living in Canada.^21^ Data for the CLSA COVID-19 study were collected from 28 559 CLSA participants (from both cohorts; 67.2% of the eligible sample) over eight months (April 2020-December 2020). Participants completed a 30-minute baseline questionnaire (COVID-B, April 2020-May 2020), four 10-minute weekly questionnaires (COVID-W, for those completing the internet survey) or two 10-minute bi-weekly questionnaires (COVID-BW, for those completing the telephone survey), three 10-minute monthly questionnaires (COVID-M) and a final 30-minute exit questionnaire (COVID-E, September 2020-December 2020) via web-questionnaire or telephone interview. The COVID-B survey collected information on COVID-19 status and symptoms, risk factors, health care usage, self-quarantine behaviour, and psychological, social, and economic changes during the pandemic. The COVID-E survey collected information on COVID-19 status and symptoms, and psychological and functional ability variables. The weekly, biweekly, and monthly questionnaires focused mainly on pandemic-related behaviours and COVID-19 status and symptoms.^21^

#### Measures

##### Change in Functional Ability During the Pandemic

Change in functional ability between the time of the COVID-B and COVID-E surveys was assessed using seven questions from the COVID-19 Aging and Mobility Survey created by the CLSA (Data Collection, 2020). This 7-question measure asked participants to report the degree to which their perceived functional ability and daily activities had changed between March 1, 2020, and the time of the exit survey (September-December 2020). These abilities and activities included moving about their house, doing chores, engaging in physical activity, keeping in touch with others, taking care of their health, running errands, participating in their community, and maintaining social connections. These questions were scored on a 5-point scale with a score of 1 indicating that their ability to perform a task was much worse, 2 indicating a little worse, 3 indicating about the same, 4 indicating a little better and 5 indicating much better. For ease of interpretation, we recentred the scale so that no change in ability (3, ‘about the same’) became 0, scores indicating improvement remained positive numbers (4 and 5 were recoded into 1 and 2, respectively) and scores indicating decline became negative numbers (1 and 2 were recoded into −2 and −1, respectively). Scores across the seven questions were summed to obtain a change in functional ability score, with negative scores indicating overall declined functional ability and positive scores indicating overall improved functional ability since the start of the COVID-19 pandemic.

##### Functional Ability at FUP1

Functional ability was assessed at follow up 1 (FUP1, 2015-2018) in the core CLSA study using a modified version of the Activities of Daily Living questions from the Older Americans Resources and Services (OARS) Multidimensional Assessment Questionnaire.^22^ This scale assesses ability to perform Basic Activities of Daily Living (BADL) including feeding and dressing oneself, walking around, and getting in and out of bed, and Instrumental Activities of Daily Living (IADL) including using the telephone, traveling, grocery shopping, and housework.

Based on their ability to perform these activities, participants were classified as having no functional impairment, mild impairment, moderate impairment, severe impairment, or total impairment. For the purpose of this analysis, functional ability was converted to a binary variable (1 = at least mild impairment [difficulty doing at least 1 BADL or IADL on their own] or, 0 = no functional impairment).

##### Cognition at FUP1

To account for cognitive status at FUP1, a composite cognitive variable was created, as described elsewhere.^23^ Briefly, raw scores on the Rey Auditory Verbal Learning Task (immediate and 5-minute delayed recall),^24^ Mental Alternation Task,^25^ and Animal Fluency Test^26^ were converted to *z*-scores and summed to create a composite score at follow-up. The composite z-score was then converted to a composite T-score using the following calculation: (composite z-score X 10) + 50. Higher T-Scores represent better performance.

##### Depressive Symptoms

Depressive symptoms were assessed using data from the CLSA FUP1 and COVID-B surveys using the Center for Epidemiological Studies Depression Scale (short form; CES-D-10).^27^ In this self-report measure participants were asked to respond to 10 questions regarding how frequently they had experienced various depressive symptoms such as being easily bothered or scared/fearful in the past seven days. Each question was rated on a 4-point scale ranging from 0-3 with a possible maximum score of 30 with higher scores indicating greater frequency of depressive symptoms. A score of 10 or higher is suggestive of “significant” depressive symptomatology. In the current analysis, we examine depressive symptoms at COVID-B, although it was also measured at COVID-E. Depressive symptoms at COVID-E, as well as change in depressive symptoms from COVID-B to COVID-E, were not examined as previously reported findings indicated little change in symptomatology across the overall sample (see Parminder et al, 2021^12^; Table S1).

##### Anxiety Symptoms

Anxiety symptoms were measured using a 7-item scale assessing generalized anxiety disorder, the GAD-7 (Generalized Anxiety Disorder-7^28^) in the COVID-B survey. This self-report measure required participants to rate, on a 4-point scale ranging from 0-3, how often they had experienced anxiety related symptoms such as feeling nervous or having difficulty relaxing over the past two weeks. The GAD-7 total score ranges from 0-21 with higher scores indicating greater frequency of anxiety symptoms. A score of 10 or greater represents a cut point for identifying GAD. In the current analysis, we examine symptoms of anxiety at COVID-B, although it was also measured at COVID-E. Anxiety symptoms at COVID-E, as well as change in anxiety symptoms from COVID-B to COVID-E, were not examined as previously reported findings indicate little change in symptomatology across the overall sample (see Parminder et al, 2021^12^; Table S1).

##### Self-Quarantining

The COVID-B, COVID-W, COVID-BW and COVID-M surveys each contained YES/NO questions regarding participant behaviours during the pandemic, including a question about self-quarantining. A full list of questions can be found within the COVID-19 survey documents on the CLSA Website (https://www.clsa-elcv.ca/researchers/data-collection). For the present study, we focused on the question about self-quarantining: ‘In the past week/two weeks/month have you been under self-quarantine, which means that you have only had physical contact with your immediate household members?’. Some of the participants received only the COVID-W survey, which was administered twice, and other participants received only the COVID-BW, which was administered four times. As such, in our analysis we only included surveys that all participants were administered, that is, the COVID-B (administered once) and COVID-M surveys (administered three times following the COVID-W and COVID-BW surveys).

The frequency at which each participant answered ‘yes’ to the self-quarantining question during the COVID-B and COVID-M surveys was summed and converted into a frequency score ranging from 0-4.

##### Demographic Covariates

Demographic variables such as age at COVID-B were collected from the CLSA COVID-19 study, whereas variables such as sex (male/female) and level of education, were extracted from the core CLSA study (Baseline). Level of education was assessed using the following options: less than secondary school graduation, secondary school graduation (no post-secondary education), some post-secondary education and post-secondary degree/diploma. For these analyses, level of education was converted to a binary variable (1 = post-secondary degree/diploma, 0 = no post-secondary degree/diploma). Living alone was extracted from the COVID-B survey. Participants were asked “how many people (including yourself) currently live in your residence? This includes people who sleep there at least 3 nights per week.” Participants who replied ‘1’ were coded as living alone (1) and participants who replied any other number were coded as not living alone (0). Additionally, participants were asked to indicate whether they had a long-term chronic health condition during the COVID-B survey, such as diabetes, cancer, etc (please see survey on the CLSA website for a complete list of conditions). We also incorporated a pain variable, which was extracted from the main CLSA study (FUP1), which asked participants to indicate whether they were usually free of pain or discomfort or not. Note that pain was not asked about in the COVID questionnaires (beyond pain associated with COVID-19 infection). Lastly, we incorporate a hearing variable using a question from the main CLSA study (FUP1), which asked participants to rate their hearing (using a hearing aid if applicable) from Excellent to Poor. We created a binary variable in which participants who rate their hearing as fair or poor were coded as having hearing issues (1) and participants who rated their hearing as good, very good or excellent were coded as hearing fine (0).

#### Statistical Analyses

First, Independent sample *t*-tests and Chi-square tests were used to compare sample characteristics between included and excluded participants.

Next, we used hierarchical linear regression to assess the effect of self-quarantining, depressive and anxiety symptoms at COVID-B, and functional and cognitive ability at FUP1 on perceived change in functional ability at COVID-E. As well, a two interaction terms were created: frequency of self-quarantining X depression at COVID-B, and frequency of self-quarantining X anxiety at COVID-B. These interaction terms were used to assess whether the relationship between self-quarantining and perceived change in functional ability differed based on levels of anxiety or depressive symptoms. Next, anxiety at COVID-B, depression at COVID-B and frequency of self-quarantining were mean-centred to reduce collinearity with the interaction terms, which were re-created using these newly mean-centred terms. Demographic variables: age, sex, years of education, living alone, and chronic condition were entered into Model 1. Variables from FUP1: pain, hearing loss, cognition, and functional ability were entered into Model 2. Variables from COVID-B: anxiety and depressive symptoms were entered into Model 3. Next, data collected over the course of the COVID-19 survey: frequency of self-quarantining, was entered into Model 4. Lastly, the two interaction terms were entered into Model 5. Significant interactions were further explored using simple slopes analysis.

Reported results include unstandardized Beta coefficient estimates with 95% Confidence Intervals (CI) for each variable entered into each model, R Square and Adjusted R Square values, as well as the F Change Statistic for each Model. Models were also assessed for multicollinearity (post-mean-centring) using the Variance Inflation Factors (VIF) and Condition Index. All analyses were conducted in IBM SPSS Statistics 27.

### Results

#### Sample Characteristics

**Figure 1.**
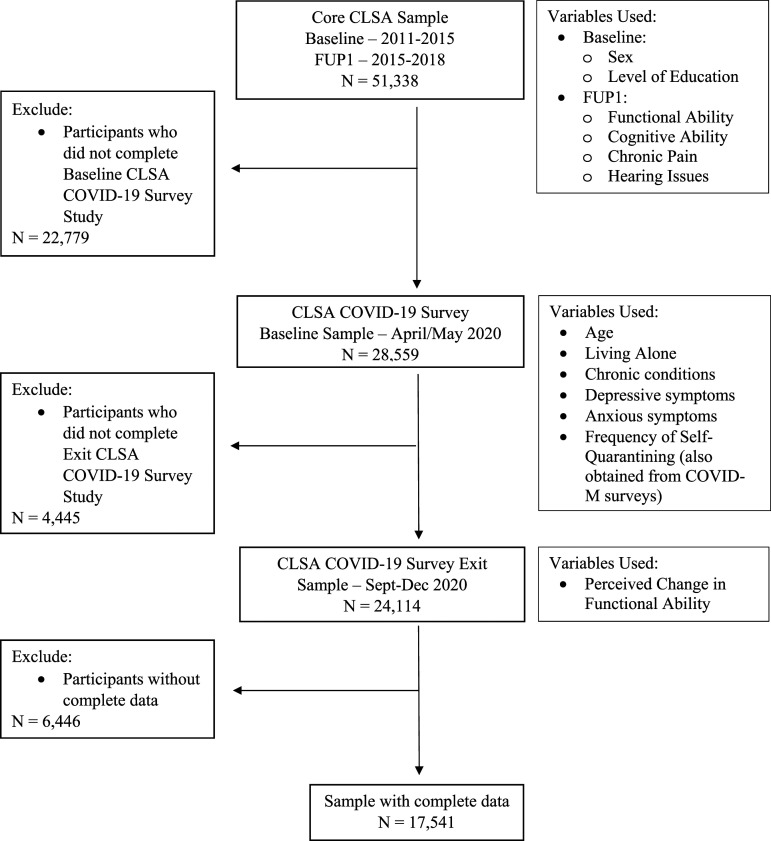
Exclsuion criteria and variables used for this study for each wave of data collections from the core CLSA and CLSA COVID-19 survey studies.

Of the 28 559 CLSA participants who completed. the COVID-19 surveys, 24 114 completed both the COVID-19 Baseline and Exit surveys, and 17 541 of those participants had complete data on all variables of interest (see Figure 1). The eligibility criteria for inclusion in this study are depicted in Figure 1. Independent samples *t*-tests revealed significant differences in age, cognition at FUP1, depression, anxiety, and frequency of self-quarantining between the included and excluded samples (see Table 1). The excluded sample was significantly older than the included sample, had lower cognitive scores, reported more depressive and anxiety symptoms, and reported self-quarantining less times on average compared to the included sample. However, the *N* for the excluded sample was significantly reduced for some variables due to missing data (see Table 1 Note; see Discussion for further interpretation). Chi Squared tests revealed no significant differences in sex distribution between the included and excluded samples; however, there was a significant difference in education, living alone and functional ability at FUP1 such that the excluded sample had a significantly lower percentage of participants with at least a post-secondary degree/diploma, a higher percentage of participants living alone as well as participants who were functionally impaired at FUP1 compared to the included sample. Please refer to Raina et al (2021)^12^ for a comparison of socio-demographic characteristics across the CLSA surveys examined in the present study (Baseline, FU1, COVID-B, COVID-E).

**Table 1.** Included (Study) Sample Versus Excluded Sample Characteristics.

	Included Sample (N = 17 541)		Excluded Sample (N = 11 024)		*t* or c^2^
	Mean or %	SD	Mean or %	SD	
Age	68.33	9.33	69.46	10.2	9.42**
Sex (% F)	52.3	—	52.72	—	.48
Education (% post-secondary degree/diploma)	79.38	—	76.64^a^	—	29.77**
Living alone (%)	23.92	—	26.37^a^	—	20.71**
Chronic condition (%)	58.11	—	60.86^a^	—	20.41**
Chronic pain (%)	32.88	—	36.88^a^	—	47.29**
Hearing issues (%)	12.5	—	15.07^a^	—	37.78**
Functional ability (FUP1; % impaired)	9.88	—	14^a^	—	106.67**
Change in functional ability during the pandemic (COVID-E)	−1.31	2.49	−1.38^a^	2.75	1.71
Cognition (FUP1; T-score composite)	52.28	28.44	46.3^a^	29.19	14.23**
Depression (COVID-B)	5.74	5.04	6.43^a^	5.34	10.51**
Anxiety (COVID-B)	2.58	3.57	2.74^a^	3.78	3.33**
Frequency of self-quarantining (based on 4 time points)	.76	0.9	0.7	.86	5.75**

Note. **P < .001. SD = standard deviation. For Sex, Education, Living Alone, Chronic Condition, Chronic Pain, Hearing Issues and Functional Ability at FUP1, numbers are displayed as percentages; for all other variables, numbers are displayed as means. Functional ability at FUP1 represents the percentage of individuals classified as at least mildly impaired by the OARS (see Methods for details). For Change in Functional Ability During the Pandemic, positive values represent perceived overall better functional ability whereas negative values represent perceived overall worse functional ability at COVID-E compared to March 1, 2020. Frequency of Self-Quarantining represents the number of times individuals self-reported that they had self-quarantined at four time points (COVID-B and the three COVID-M surveys).

^a^Due to missing data, the *N*’s were less than the entire excluded sample for these variables. Education: N = 10 969; Living Alone: N = 10 135; *Functional* Ability: N = 9876; Change in Functional Ability: N = 5616; Cognition: N = 6540; Depression: N = 9930; Anxiety: N = 8338.

Sample characteristics of the included and excluded samples are displayed in Table 1. The included sample was quite even in sex distribution and the majority of the sample had a post-secondary degree or diploma. Less than one-quarter of participants reported living alone and only a small proportion of the sample (9.88%) reported being functionally impaired at FUP1. The sample reported an overall worsening in perceived functional ability since the start of the pandemic *(M =* −1.31; a negative score indicates perceived worse functional ability). Overall, cognition at FUP1 was within the average range (T Score = 52.3). There were generally low frequencies of self-quarantining (*M* = .76, range = 0-4).

#### Functional Ability Analyses

The complete results of the linear regression analyses are displayed in Table 2 (see Table S1 for 95% Confidence Intervals). After mean-centring, the VIF remained below 10 and Condition Index remained below 30 for all models, suggesting acceptable levels of multicollinearity.

**Table 2.** Hierarchical Linear Regression Analysis of Predictors of Change in Perceived Functional Ability.

Predictor Variables	Model 1		Model 2		Model 3		Model 4		Model 5	
	B	β	B	β	B	β	B	β	B	β
Block 1										
Age	−.008**	−.03	−.006*	−.022	−.017**	−.063	−.015**	−.058	−.016**	−.058
Sex (female)	−.29**	−.059	−.21**	−.043	−.053	−.011	−.046	−.009	−.046	−.009
Education (post-secondary degree or diploma)	−.21**	−.035	−.23**	−.037	−.22**	−.036	−.23**	−.037	−.22**	−.036
Living alone	−.13**	−.022	−.1*	−.017	.058	.01	.056	.01	.053	.009
Chronic condition	−.35**	−.069	−.26**	−.052	−.2**	−.039	−.19**	−.037	−.19**	−.037
Block 2										
Chronic pain			−.41**	−.077	−.24**	−.045	−.24**	−.045	−.24**	−.044
Hearing issues			−.41**	−.055	−.27**	−.035	−.27**	−.035	−.26**	−.035
Cognition (FUP1)			−.002*	−.026	−.003**	−.037	−.003**	−.037	−.003**	−.036
Functional status (FUP1)			−.71**	−.085	−.51**	−.06	−.49**	−.058	−.48**	−.058
Block 3										
Depression (COVID-B)					−.11**	−.22	−.11**	−.22	−.11**	−.22
Anxiety (COVID-B)					−.025**	−.035	−.025**	−.035	−.024**	−.034
Block 4										
Frequency of self-quarantining							−.1**	−.037	−.098**	−.035
Block 5										
Frequency of self-quarantining X depression									−.013*	−.025
Frequency of self-quarantining X anxiety									−.009	−.012
Adjusted *R*^2^	.011		.029		.086		.087		.088	
Δ*R*^*2*^	.011		.018		.057		.001		.001	
Δ*F*	40.68		82		547.66		24.82		11.52	

*Note.* *P < .05, **P < .01. B values displayed are unstandardized beta coefficients. β values displayed are standardized beta coefficients.

##### Models 1-3: Demographics, Cognition, Functional Ability & Mental Health

All demographic variables entered in Model 1 were statistically significant such that older age, female sex, having more than 12 years of education, living alone, and having at least 1 chronic condition were associated with perceived decreases in functional ability. Chronic pain, hearing issues, higher cognitive ability, and impaired functional ability at FUP1 were statistically significant predictors of perceived decrease in functional ability at COVID-E in Model 2. Greater levels of anxiety and depressive symptoms at COVID-B were also significant predictors of perceived decrease in functional ability at COVID-E in Model 3. After the addition of variables in Model 3, female sex and living alone were no longer significant predictors in the model. Blocks 1-3 combined accounted for 8.2% of the variance in perceived change in functional ability at COVID-E; *F* (8, 17 659) = 198.06, *P* < .001.

##### Models 4-5 Including Frequency of Self-Quarantining

In Model 4, greater frequency of engaging in self-quarantining was associated with a perceived decrease in functional ability at COVID-E, although this behaviour only accounted for .1% additional variance in the model (B = −.11, *t* = −5.56, *P* < .001). Finally, in Model 5, the interaction between self-quarantining and depressive symptoms was significant. A subsequent simple slopes analysis revealed that the negative relationship between self-quarantining and functional ability (i.e., more frequent self-quarantining = perceived worsening functional ability) was significant only at average and high levels of depressive symptoms at COVID-B. The interaction between self-quarantining and levels of anxiety at COVID-B was not significant in the model. The final model accounted for 8.5% of the variance in perceived change in functional ability at COVID-E; *F* (11, 17 656) = 149.34, *P* < .001. Effect sizes (β weights) for all models were minuscule-small.

### Discussion

Our primary aim was to determine if pre-pandemic cognitive and functional ability as well as mental health symptoms and self-quarantining during the pandemic were predictive of changes in perceived functional ability in older Canadians during the COVID-19 pandemic. The results of our study are meaningful in that they identify key predictors of perceived functional ability among older adults during the COVID-19 pandemic, which may be considered during future pandemics or situations requiring social distancing behaviours. Our results demonstrate support for some of our hypotheses.

Our hypotheses that lower levels of cognitive ability at FUP1 of the core CLSA study would be associated with perceived decreases in functional ability during the pandemic was not supported. On the contrary, higher levels of pre-pandemic cognitive ability were associated with decreases in perceived functional ability over the first 6-9 months of the pandemic. Conversely, however, we did find support for our hypothesis that lower levels of reported functional ability prior to the pandemic would be associated with perceived loss of functional ability during the pandemic. A possible explanation for these findings is that participants who were more cognitively healthy prior to the pandemic may have begun to require more support over the course of the pandemic and as a result perceived being less functionally able at the exit survey compared to their pre-pandemic functional ability. In other words, individuals with lower cognitive ability may have already been more dependent at the start of the pandemic, and as such were less likely to perceive further declines, whereas cognitively healthier individuals perceived a greater loss in functional ability compared to their pre-pandemic state. This finding has implications for health care providers and caregivers who are working with older adults during the pandemic, as older adults with greater cognitive ability may be at some greater risk for noticeable declines in functional ability over the course of the pandemic, or more likely to report requiring assistance. However, without data on objective cognitive performance, or IADLs or ADLs during the pandemic, we cannot be certain whether concurrent objective cognitive and/or functional ability may be associated with perceived change in functional ability over the study period.

As expected, greater levels of anxiety and depressive symptoms reported at COVID-B were associated with greater perceived decline in functional ability. These results are consistent with previous research indicating that depressive and anxiety symptoms negatively affect older adults’ ability to function independently.^13,29,30^ The addition of depressive and anxiety symptoms to the model also caused female sex and living alone status to no longer predict declines in perceived functional ability. Thus, anxiety and depressive symptoms share a large degree of variance with these demographic variables. Healthcare providers and caregivers of older adults during the pandemic should monitor the mental health of older adults as the pandemic progresses; level of depressive symptoms later in the pandemic appear to be more indicative of perceived loss of functional ability compared to level of depressive symptoms prior to the pandemic.

We also found that lower frequency of engaging in self-quarantine behaviour was associated with perceived increases in functional ability over the course of the pandemic. Thus, not isolating oneself indoors appeared to have a positive impact on functional ability despite the possible increased risk of contracting COVID-19. This is consistent with previous findings indicating that decreased activity and increased sedentary behaviours due to the pandemic have led to reduced functional ability in older adults.^10^ However, it is important to distinguish between the statistical and practical significance of these results as self-quarantining accounted for less than 1% of the variance in our model. Thus, it is unlikely that frequency of self-quarantining meaningfully contributed to one’s perceived change in functional ability during the pandemic despite our statistically significant findings. In line with this, the effect sizes seen across tested models ranged from miniscule to small suggesting that while statistically significant, the relationships observed may not be to an extent that would be considered clinically significant for most older adults. These findings may help to inform policy makers who are making decisions regarding social gatherings and isolation requirements during the pandemic as the results from this study suggest that engaging in self-isolating behaviours does not greatly impact the perceived functional ability of older adults.

#### Strengths and Limitations

A primary strength of our study is the large sample representative of older adults across Canada from the CLSA. Additionally, the use of this longitudinal data allowed us to examine change in depression and cognitive ability prior to the start of the pandemic.

Despite these strengths, this study had several limitations, a primary limitation being that not all variables were collected at all time points. Had functional ability been measured during the weekly, biweekly, and monthly COVID-19 surveys, coupling models may have been used to determine if the timing of self-quarantining had an impact on perceived changes in functional ability. Additionally, it is unclear whether perceived changes in functional ability on the COVID-E survey represent true changes in functional ability or whether they represent lack of ability to partake in activities due to pandemic-related restrictions. Unfortunately, objective measures of functional ability were not available for this study. Data regarding premorbid levels of depression and anxiety or mental health diagnoses immediately prior to the pandemic may have increased insight into the mental health of participants leading into the pandemic as the data from FUP1 were collected several years prior to the pandemic. Similarly, while cognitive variables from the core CLSA study were used in this study, no cognitive data were collected during the CLSA COVID-19 study, and as such there may have been changes in cognitive status between the FUP1 and the CLSA COVID-19 study that were unaccounted for. Such changes in cognitive status may have been associated with perceived changes in functional status during the pandemic despite the lack of association between change in pre-pandemic cognitive function and functional ability seen in this study. However, adding these variables to the CLSA COVID-19 study would have been very demanding for participants and potentially difficult to collect via telephone and video conferencing. Still, such limitations should be considered in future studies. Lastly, there were differences between the included and excluded samples across several variables including demographics such as age and education, as well as functional ability at FUP1, cognition, depression, anxiety, and self-quarantining. However, while the excluded sample had a higher percentage of individuals who were at least mildly functionally impaired, there were no differences between the two samples in change in functional impairment. Further, despite cognition being lower in the excluded sample, it remained within the Average range. Likewise, while absolute levels of depression and anxiety were slightly higher in the excluded sample, scores on these measures remained generally not clinically significant, within the mild and minimal ranges, respectively, as was the case for the included sample.

Additionally, we were unable to account for temporal effects. It is possible that these perceived changes in functional ability since the start of the pandemic (a six-to 9-month period depending on when COVID-B was first administered) may have occurred regardless of the COVID-19 pandemic. However, given the evidence of the negative effect of the pandemic on older adults’ perceived functional ability,^8,10^ it is unlikely that these changes are unrelated to the pandemic. Lastly, it should be noted that there was a higher percentage of excluded participants who reported at least mildly impaired functional ability at FU1. This suggests that those who did not participate in the COVID surveys were more likely to have lower functional ability.

#### Conclusions and Future Directions

Overall, the results from this study suggest that older adults with higher levels of cognitive ability but lower levels of functional ability prior to the onset of the pandemic may be at increased risk for perceived decreases in functional independence as pandemics arise, making those with preserved cognitive abilities a population of interest in future studies. Additionally, higher levels of anxiety and depressive symptoms reported during the pandemic (between September and December 2020) were associated with concomitant decreased perceived functional ability during this period. These findings have implications for policy makers who implement pandemic-related restrictions and recommendations while considering the cognitive and mental health, and functional independence, of older adults in Canada. These results also have implications for clinicians who may be working with older adults during the pandemic in the hopes of improving their mental health and maintaining their functional ability. Further research should be conducted to replicate the findings of this study and investigate whether physically distanced forms of communication (e.g., video calls) have similar associations with changes in perceived functional ability as do in-person behaviours dependent on leaving the house, and whether ability to access such measures is impacted by level of cognitive ability.

## Supplemental Material

Supplemental Material - The Effects of Cognitive Ability, Mental Health, and Self-Quarantining on Functional Ability of Older Adults During the COVID-19 Pandemic: Results From the Canadian Longitudinal Study on Aging Supplemental Material for The Effects of Cognitive Ability, Mental Health, and Self-Quarantining on Functional Ability of Older Adults During the COVID-19 Pandemic: Results From the Canadian Longitudinal Study on Aging by Madeline A. Gregory, Morgan J. Schaeffer, Jennifer T. H. Reeves, Lauren E. Griffith, Christina Wolfson, Nicole E. Basta, Jacqueline M. McMillan, Susan Kirkland, Parminder Raina, and Theone S. E. Paterson, on behalf of the Canadian Longitudinal Study on Aging (CLSA) Team in Journal of Geriatric Psychiatry and Neurology.

## Data Availability

Data are available from the Canadian Longitudinal Study on Aging (www.clsa-elcv.ca) for researchers who meet the criteria for access to de-identified CLSA data.^31^
