# Supplementary material for: The Effects of Cognitive Ability, Mental Health, and Self-Quarantining on Functional Ability of Older Adults During the COVID-19 Pandemic: Results From the Canadian Longitudinal Study on Aging
Source: J Geriatr Psychiatry Neurol. 2023 Dec 20;37(4):307–17. doi: 10.1177/08919887231218755 (PMC11089823; doi:10.1177/08919887231218755)
Supplement: Supplemental Material - The Effects of Cognitive Ability, Mental Health, and Self-Quarantining on Functional Ability of Older Adults During the COVID-19 Pandemic: Results From the Canadian Longitudinal Study on Aging [file sj-pdf-1-jgp-10.1177_08919887231218755.pdf]

Table S1. Nested Models Analysis of Predictors of Change in Perceived Functional Ability Using Confidence Intervals

	Model 1		Model 2		Model 3		Model 4		Model 5	
Predictor Variables	B	95% CI	B	95% CI	B	95% CI	B	95% CI	B	95% CI
**Block 1**										
Age	**-0.008**	-0.12<br>-0.004	**-0.006**	-0.01<br>-0.001	**-0.017**	-0.021<br>-0.3	**-0.015**	-0.02<br>-0.011	**-0.016**	-0.02<br>-0.011
Sex (female)	**-0.29**	-0.37<br>-0.22	**-0.21**	-0.29<br>-0.14	-0.053	-0.13<br>0.022	-0.046	-0.12<br>0.029	-0.046	-0.12<br>0.029
Education (post-secondary degree/diploma)	**-0.21**	-0.31<br>-0.12	**-0.23**	-0.32<br>-0.13	**-0.22**	-0.31<br>-0.13	**-0.23**	-0.32<br>-0.14	**-0.22**	-0.31<br>-0.14
Living alone	**-0.13**	-0.22<br>-0.04	**-0.098**	-0.19<br>-0.009	0.058	-0.029<br>0.15	0.056	-0.031<br>0.14	0.053	-0.034<br>0.14
Chronic Condition	**-0.35**	-0.43<br>-0.27	**-0.26**	-0.34<br>-0.19	**-0.2**	-0.27<br>-0.13	**-0.19**	-0.26<br>-0.11	**-0.19**	-0.26<br>-0.11
**Block 2**										
Chronic Pain			**-0.41**	-0.49<br>-0.33	**-0.24**	-0.32<br>-0.16	**-0.24**	-0.31<br>-0.16	**-0.24**	-0.31<br>-0.16
Hearing Issues			**-0.41**	-0.52<br>-0.3	**-0.27**	-0.38<br>-0.16	**-0.27**	-0.37<br>-0.16	**-0.26**	-0.37<br>-0.15
Cognition (FUP1)			**-0.002**	-0.004<br>-0.001	**-0.003**	-0.005<br>-0.002	**-0.003**	-0.005<br>-0.002	**-0.003**	-0.005<br>-0.002
Functional Status (FUP1)			**-0.71**	-0.84<br>-0.58	**-0.51**	-0.63<br>-0.38	**-0.49**	-0.61<br>-0.36	**-0.48**	-0.61<br>-0.36
**Block 3**										
Depression (COVID-B)					**-0.11**	-0.12<br>-0.1	**-0.11**	-0.12<br>-0.1	**-0.11**	-0.12<br>-0.099
Anxiety (COVID-B)					**-0.025**	-0.039<br>-0.01	**-0.025**	-0.039<br>-0.01	**-0.024**	-0.038<br>-0.01
**Block 4**										
Frequency of Self-Quarantining							**-0.1**	-0.14<br>-0.062	**-0.098**	-0.14<br>-0.058
**Block 5**										
Frequency of Self-Quarantining X Depression									**-0.013**	-0.024<br>-0.002
Frequency of Self-Quarantining X Anxiety									-0.009	-0.024<br>0.006
Adjusted R^2	.011		.029		.086		.087		.088	
ΔR^2	.011		.018		.057		.001		.001	
ΔF	**40.68**		**82**		**547.66**		**24.82**		**11.52**	

Note. B values displayed are unstandardized beta coefficients. 95% Confidence Intervals (CI) are reported as lower bound values followed by upper bound values. Bolded B and F Change values are significant. β values displayed are standardized beta coefficients.
